# Supplementary material for: In the quest for effective factors of satisfaction with life: Insights from intra-couple interaction and financial management variables
Source: PLoS One. 2023 Mar 16;18(3):e0279079. doi: 10.1371/journal.pone.0279079 (PMC10019632; doi:10.1371/journal.pone.0279079)
Supplement: Attachment — Submitted filename: Response to Reviewers.docx [file pone.0279079.s001.docx]

Dear Reviewer,

Thank you for the opportunity to revise our paper on ‘In the quest for effective factors of relationship quality: Insights from intra-couple interaction and financial management variables’. Your suggestions have been very helpful for improving the manuscript.

I have included Your comment after this letter and responded to indicating how we addressed Your suggestion and describing the changes we have made. Due to the suggestion to change the statistical strategy, we decided to enrich our team with an expert in the area. The revisions have been approved by all authors. The changes are marked in the ‘track changes’ mode in the paper.

We hope the revised manuscript will better suit and we thank you for your continued interest in our research.

Kind regards,

Monika Baryła-Matejczuk

Response to **Reviewer #1** Comments

**Point 1:** This paper describes a study investigating the associations among financial management behaviours and relationship quality in heterosexual couples in Poland. The introduction needs to be "smoothed" out with less repetition. I suggest beginning with the theory, then provide a literature that does or does not report the theory and then what this study will show about the theory.

**Response 1:** Thank you for your suggestions to improve this manuscript. The theoretical part has been rebuilt, the introductions have been shortened in order to keep the essence, the theoretical basis begins the article, successively reports from the literature and research were recalled explaining the direction taken in the prepared analyses.

**Point 2:** My major concerns are with the analyses:

**2.1** A smaller concern is that I could not figure out what the instrument was for relationship quality, that needs to be made clearer, what were the items?

**Response 2.1:** Thank you for your comment, the Well-Matched Marriage (KDM-2) questionnaire was used to measure the quality of the relationship (source: Kaźmierczak M, Plopa M. Communication in Marriage Questionnaire – Conclusions from Marital Studies. Pol Forum Psychol. 2006; 11(2): 213-226.). The questionnaire has a four dimensions: intimacy, disappointment, self-realization, and similarity as well as the overall result indicating overall quality of relationship. In our analysis, we took into account the overall result. Questions that go into the tool include i.a.:

Marriage / relationship life with its struggles is only the authentic life that every human being should experience.

In our free time, we try to be together.

As the years go by, the contact between us deepens and we feel more and more connected with each other.

I regret the lost independence, freedom from the pre-marriage period.

When we have a sharp exchange of views, this fact causes a long-lasting disconnect.

We agree when it comes to spending free time, weekend and holidays.

As the marriage / relationship lasts, we are more sensitive to our own needs, more subtle, mature in mutual contacts.

Relationship / marriage has become an obstacle for me in achieving my own goals, for example professional aspirations.

Joint activities and the implementation of joint plans bring me satisfaction.

I feel better at work than at home.

I can say that I found happiness in marriage / happiness in a relationship.

I feel lonely in a marriage / relationship.

Our worldview coincides.

As the examples above shows, the questionnaire consists of statements about marriage / relationship, some of them refer directly to a person as a partner in a marriage / relationship, while others refer to marriage / relationship as a whole.

**2.2** Although it was appropriate to separate the males and females given the nonindependence of the data and adding the other partner's satisfaction was important, I think a dyadic data analysis process would have been more appropriate. I was also missing a correlation table showing the correlations of the variables both within and between partners. I worry that the "flip" in the direction of the relationships is more about multicolinearity than reality. Cook and Kenny have shown these "flips" when including multiple family members' data in the same regression equation.

**2.3** I don't understand why an Actor Partner Interdependence model was not used. In the APIM both partner's data and both partner's outcomes are included in the model and actor and partner paths can be tested for equivalence. Using step wise methods are problematic for testing theory, and I don't think it is appropriate to discuss gender differences without also testing the estimates for equivalence. I think the APIM is a much more appropriate model to test and would provide a better test of the theory, than what was presented here.

**Response 2.2&2.3:** Thank you for the suggestion of improvement and the opportunity to refer readers to other studies. We have built two APIM models as presented by Cook and Kenny. The result of the chi-squared test (chi^2(12) = 24.039, p = 0.02) enables us to reject the hypothesis of actors indistinguishability. Therefore, the model proposing the distinguishability with respect to gender has been further discussed in the manuscript. Thanks to this great suggestion, we were able to identify both actor and partner effects. As a consequence sections: Analysis, Results and Discussion have been modified accordingly.

Unfortunately, no one at our research team had experience with APIM models. We therefore decided to invite an experienced researcher to help us prepare APIM models. After more than a dozen attempts, we were able to estimate two models that shed new light on the relationships analysed in the text. However, there was a change in the independent variables. The harsh start-up variable spoiled all the models, so we decided to replace it with a well-being variable, which (according to the theoretical approach) very often appears in analyses of different couple relationships. All the changes made have been marked accordingly in the text.

**2.4** Given the fact that we don't know if the differences between the equation for males and equation for females were statistically significant, and issues with multicollinearity, I don't know that I would trust the discussion section. I would rather see an APIM model with the actor and partner effects tested for equivalence between the partners, before I would be able to trust that the gender differences shown were actual differences and not artifacts of the analyses.

**Response 2.4** The discussion section has been reworked based on data from the APIM model. The results shed new light on the analysed relations and constitute material worth extending with further research. The results obtained with the APIM model were presented in the Results section, and their interpretation appeared in the Discussion section.
