# Supplementary material for: In the quest for effective factors of satisfaction with life: Insights from intra-couple interaction and financial management variables
Source: PLoS One. 2023 Mar 16;18(3):e0279079. doi: 10.1371/journal.pone.0279079 (PMC10019632; doi:10.1371/journal.pone.0279079)
Supplement: Attachment — Submitted filename: Review_response_FINAL.docx [file pone.0279079.s002.docx]

Dear Editors and Reviewers,

Thank you for the opportunity to revise our paper on ‘In the quest for effective factors of relationship quality: Insights from intra-couple interaction and financial management variables’. Your suggestions have been very helpful for improving the manuscript.

The next round of reviews gave us the impetus to make the essential changes to the text, which we hope have now put our proposal at a very high level. Unfortunately, in the course of working on the next version of the APIM model, we discovered a mistake in the code that put everything we had prepared so far into question. In one line of code, we discovered an unwanted variable, the presence of which affected the quality of the model and, as a result, the value of the ANOVA test. After removing the unwanted variable, the model turned out to be indistinguishable, marking an 180-degree turnaround from the previous (first) version and most importantly, our knowledge of the issue and the theoretical foundations did not allow us to accept this solution. The easiest solution would be to quit and forget, but as experienced researchers we decided to save the project and valuable time of reviewers that has been invested into our manuscript. After discovering a mistake in the code that turned our work upside down we decided to return to the idea that guided us at the beginning of designing the research and writing the text. We changed the dependent variable KDM to well-being, and further enriched the entire model with the variable harsh start-up. That was the original assumption that proved correct at this stage of review.

We are aware that such things practically do not happen at this stage of the review, but in keeping with the principle of honesty, we decided to inform you about the problems and analytical strategy we have chosen. What could we add?

1. APIM was very new analytical model that we have learnt during the review process. Despite the some great articles describing the use of this approach, translating it into programming code was quite a challenge for us. We decided to invite an additional analyst to cooperate with us, who would be able to prepare the model. The cooperation of two analysts allowed to discover the error in the code.

2. We decided to openly and honestly tell about what happened to us, because we believe that the work of a scientist is often unpredictable, surprising, but also very devastating. You can accuse us of inaccurate preparation of the model after the first wave of reviews, but we emphasize that we get familiar with APIM relatively recently.

3. The model we have prepared now is the result of several dozen hours of work, a choice among several dozen other models, which may prove that it can contribute a lot to widely understood social science.

Despite the major change we outlined, we are happy that we discovered the error found at this stage. There would be nothing worse than publishing a text that presented the results of an incorrectly built model.

Kind regards,

Monika Baryła-Matejczuk

Response to Reviewer #1:

The authors have done a great job responding to the original review. I appreciate their finding an expert in dyadic data analysis and testing for indistinguishability. There are also tests that can be performed on the paths to determine if they are statistically different, but I can let that go in this version.

**Response:** Thank you very much for the time and commitment put into the preparation of the first and second reviews for our text. We greatly appreciate the effort and try our best to live up to your expectations.

Response to Reviewer #2:

The investigators have built two APIM models as presented by Cook and Kenny with rejection of the hypothesis of actors indistinguishability. Assuming the accuracy of the fit as the investigators note, the results are very brief and descriptive and appear to follow from the modeling.

**Response:** Thank you for all the comments that allowed us to improve the text. It has been a long road to get it to a version that is in line with our research experience and the high standards of a scientific paper. The revised text is the result of our work. Notes on sampling have been added to the text and limitations.

Response to Reviewer #3:

Point 1 – Research question and scope

"Do financial management behaviors of one partner have an impact on the perception of relationship quality by the other?" I'm not sure this research question can be answered by the current design since causality cannot be inferred. For example, the current research would yield the same results with reverse causality - if one feels that their spouse perceives their relationship to be low quality (i.e., they might break up), then they might alter their financial management behavior (e.g., because the future is uncertain). Therefore, what would happen to the relationship quality if a partner improved their financial management is unclear. In fact, the authors are aware of that when they describe the "feedback loop" in line 85. However, the lack of causality here also allows for other factors to be at play. For instance, if one spouse feels unwell, that might affect both partners' financial behavior and perception of relationship quality, which would confound the analysis. Thus, the authors address the following question "Do financial management behaviors of one partner correlate with the perception of relationship quality by the other?" One way to achieve causality could be to randomize feedback for financial management – i.e., participants describe their own and partners' financial behavior, then they receive feedback that is either positive or negative (randomly), then they report relationship quality.

**Response:** thank you very much, the text has been corrected according to the note. Attention is drawn to associated than correlated.

Point 2 – Data access

(<https://data.mendeley.com/datasets/wbbgngvdvr>, the DOI link is broken for me)
Since the study is published in English, I think it will be helpful for other researchers to have the data translated. The polish version should be kept, but variable names and question translations are necessary to understand the data.

**Response:** thank you for your comments, indeed, a much greater usability of the database is possible thanks to the translation. We have translated the database and upload the current data:

Baryła-Matejczuk, Monika (2022), “Factors of relationship quality/SWLS ”, Mendeley Data, V1, doi: 10.17632/zr8tnvk43m.1

Point 3 – Measures of relationship quality

It would be interesting to see measures of relationship quality beyond the perception questionnaire. For example, how do financial management behaviors correlate with the probability of divorce? With the likelihood of having children? Tracing the participants for a follow-up survey could answer these questions and test the correlations over time.

**Response:** Thank you very much for this comment, it is an interesting and enriching remark. We will certainly consider in future research. Certainly, the limitation of the study of couples is the fact that they are declarations, self-reports. However, it would be worth considering the changes over the years. Planning longitudinal studies would also be valuable from our point of view.

Point 4 – Sample

Five hundred couples answered the survey, but how many were contacted and didn't complete it? Even if it's a representative sample demographically, attrition could create a biased sample, making it useful to report.

**Response:** Thank you for you valuable comment. In order to avoid misunderstandings we added the paragraph explaining the limitations of our sample. Representativeness could not be obtained in the study because our sample was not random. In selecting the sample, we started with the sample provided by the Central Statistical Office, which reflects a distribution of main characteristics of Polish population in terms of age / gender / voivodeship / town size. Our respondents took part in the Online Survey via the CAWI panel of the DRB Polonia company. Company controlled the distribution of those characteristics in selected sample.

„Due to the specific population of respondents we were not able to establish a sampling frame from which a random sample could be drawn. Consequently, our sample is pursposive not random. Although the purposive sampling does not yield the representativeness of results that could be projected to the population of dating couples in Poland, there are research showing that the non-probabilistic methods have similar levels of accuracy as probabilistic ones (Martinsson et al., 2013).”

Point 5 – Imputation

"The weighted predictive mean matching (midastouch) returned the lowest coefficient of variations." It is not clear to me that imputations should minimize variations. Instead, a good way to test the performance of the imputation models is to use some of the non-missing data as a test group. That is, say there are 100 complete observations (I didn't find the number of observations with missing values?), use 80 to train the model to predict each of the missing variables, then see what it predicts for the 20 observations left out. Finally, evaluate the model's accuracy (AUC/ROC for continuous, precision/recall for discrete) by comparing the test prediction with the actual observed values.

**Response:** Thank you for this comment and suggestion. We will probably turn to AI methods when imputing data in the future, considering all pros and cons of ML.

The coefficient of variation measure has been chosen as a comparison method to preserve the joint and marginal distributions for both original and imputed sample. However, this measure is not well-recognized in the literature therefore we decided to apply the RMSE (root mean square error) to evaluate our models. We had missing data in 8 variables (8% of all answers).

Here are the results of RMSE for observed vs. imputed data for 8 variables.

|  | V1 | V2 | V3 | V4 | V5 | V6 | V7 | V8 | AVG. RMSE |
| --- | --- | --- | --- | --- | --- | --- | --- | --- | --- |
| Midas | 5,051 | 5,287 | 9,433 | 9,118 | 12,234 | 12,805 | 5,567 | 5,614 | **8,139** |
| Rforest | 5,026 | 5,205 | 9,349 | 9,160 | 12,212 | 12,989 | 5,637 | 5,666 | 8,156 |
| POLR | 5,133 | 5,391 | 10,476 | 9,696 | 15,291 | 15,272 | 5,570 | 7,271 | 9,263 |

We did not insert this result to the manuscript due to the length of the article limitations. However, we decided to include this table in the answer to show that the imputation was done accurately and in accordance with the current state of knowledge in the field.

Point 9 – Analyses explanation

For a non-APIM expert audience, the analysis is tough to follow. I think there should be some explanation (a paragraph?) of the models' math/statistics and intuition. For example, it's unclear why only these five variables were included in the models (especially the interaction between well-being and shared values). What was the test for which you reported the chi-square comparing exactly (the parameters of the two models?)? What are k and the other model parameters you refer to?

**Response:** Thank you for your comment. We agree that a few extra sentences about the model will make the text more reader-friendly. We added the following part:

“To meet this goal we specified a path model in which quality of relationship reported by male and female partner were predicted simultaneously by each actor’s and hers/his partner’s: KDM, hared Goals and Values, Harsh Startup; four financial management behavior variables: Cash Management, Savings and Investment, Credit Management, and Insurance. (…) Important question regarding this type of models is whether actors within a dyad may be considered indistinguishable, in our case with respect to gender. That means, whether determinants of well-being are the same, and their effects are of the same size, for both men and women. To test this assumption, we estimated two variants of our model: in one we imposed constraints on model parameters to hold values of analogous regression coefficients in the equation for men and the equation for women the same. In other one we enabled parameters to vary between actors of different gender.”

Point 10 – Descriptive analysis

It'd be helpful to see summary statistics about each variable used in the model and their intercorrelations. That will allow understanding of collinearities and interpretation of the main results (e.g., is an effect of 1.8 large?).

**Response:** Thank you. We added two correlations matrices to Appednix. We constructed two correlation matrices for Men and Women. However, we also tested our models with a Variance Inflation Factor (VIF) to make sure there is no multicollinearity. Here are score for models: Men, Women. None of the variables go beyond level 5, which means that the problem of collinearity does not occur here.

This is what we added to Appendix:

| VIF results for two models (Men, Women) | | | | | | |  |
| --- | --- | --- | --- | --- | --- | --- | --- |
| KDM_M | SGV_M | Save_Inv_M | Harsh_M | Insurance_M | Credit_Man_M | Cash_Man_M |  |
| 1.027307 | 1.796278 | 1.910548 | 1.264026 | 1.528631 | 1.199873 | 2.019205 |  |
|  |  |  |  |  |  |  |  |
| KDM_K | SGV_K | Save_Inv_K | Harsh_K | Insurance_K | Credit_Man_K | Cash_Man_K |  |
| 1.029460 | 1.394820 | 1.453528 | 1.347075 | 1.409441 | 1.205404 | 1.234295 |  |
|  |  |  |  |  |  |  |  |
|  |  |  |  |  |  |  |  |
| Corellation matrices for two models (Men, Women) | | | | | | | |
|  | KDM_M | SGV_M | Save_Inv_M | Harsh_M | Insurance_M | Credit_Man_M | Cash_Man_M |
| KDM_M | 1.00 | -0.07 | 0.08 | 0.09 | -0.01 | 0.07 | 0.00 |
| SGV_M | -0.07 | 1.00 | 0.23 | -0.43 | 0.30 | 0.15 | 0.59 |
| Save_Inv_M | 0.08 | 0.23 | 1.00 | -0.24 | 0.55 | 0.39 | 0.52 |
| Harsh_M | 0.09 | -0.43 | -0.24 | 1.00 | -0.21 | -0.08 | -0.29 |
| Insurance_M | -0.01 | 0.30 | 0.55 | -0.21 | 1.00 | 0.30 | 0.40 |
| Credit_Man_M | 0.07 | 0.15 | 0.39 | -0.08 | 0.30 | 1.00 | 0.24 |
| Cash_Man_M | 0.00 | 0.59 | 0.52 | -0.29 | 0.40 | 0.24 | 1.00 |
|  |  |  |  |  |  |  |  |
|  | KDM_K | SGV_K | Save_Inv_K | Harsh_K | Insurance_K | Credit_Man_K | Cash_Man_K |
| KDM_W | 1.00 | -0.08 | 0.05 | 0.08 | -0.01 | 0.06 | 0.10 |
| SGV_W | -0.08 | 1.00 | 0.14 | -0.47 | 0.28 | 0.07 | 0.23 |
| Save_Inv_W | 0.05 | 0.14 | 1.00 | -0.22 | 0.44 | 0.34 | 0.36 |
| Harsh_W | 0.08 | -0.47 | -0.22 | 1.00 | -0.18 | 0.02 | -0.15 |
| Insurance_W | -0.01 | 0.28 | 0.44 | -0.18 | 1.00 | 0.32 | 0.30 |
| Credit_Man_W | 0.06 | 0.07 | 0.34 | 0.02 | 0.32 | 1.00 | 0.20 |
| Cash_Man_W | 0.10 | 0.23 | 0.36 | -0.15 | 0.30 | 0.20 | 1.00 |

Point 11 – Main results

Suppose the model's assumptions and parameters are accurate. The results show that both partners report higher relationship quality when women's cash management questionnaire scores increase. When women's insurance and shared goals and values questionnaire scores increase, their male partner weakly reports lower relationship quality. The effect of the other 20 variables was indistinguishable from zero. Also, I didn't understand how the interactional values arguments (lines 339-340) relate to the discussion, nor the size of the effect and its significance reported.

Also, I think Table 2 should include the number of observations

**Response:** Thank you for a comment. As wrote in the beginning, the whole model mode rebuilt. Indeed, lines 339-340 were redundant, therefore we removed them from manuscript.

Point 12 – Conclusions

"Our results, seen from this perspective, may suggest that couples in Poland appreciate a division of financial labour." It might be better to ask that directly in the questionnaire because this does not seem to be a plausible interpretation of the positive parameters for the woman's cash management (most other financial parameters were statistically equal to zero).
"… whereby women deal with current financial affairs, while men are authorized to handle strategic ones." what evidence in the data supports that claim? The only related evidence provided is that when women score higher on the cash management questionnaire (for which I didn't see the questions), both partners report higher marriage quality.

*A few typos I found:

Line 27: "… man's cash management predicts changes in her assessment…"

Line 62: "… pursued hereDo…"

Line 67: "Second, we explore this link using dyadic data, which creates an opportunity to compare reports of both partners.. Fourth,"

**Response:** Thank you very much for your comments, the text of the article has been corrected.
